# Mitochondrial Cardiomyopathies

**DOI:** 10.3389/fcvm.2016.00025

**Published:** 2016-07-25

**Authors:** Ayman W. El-Hattab, Fernando Scaglia

**Affiliations:** ^1^Division of Clinical Genetics and Metabolic Disorders, Department of Pediatrics, Tawam Hospital, Al-Ain, United Arab Emirates; ^2^Department of Molecular and Human Genetics, Baylor College of Medicine, Houston, TX, USA

**Keywords:** hypertrophic cardiomyopathy, dilated cardiomyopathy, restrictive cardiomyopathy, non-compaction cardiomyopathy, histiocytoid cardiomyopathies, Barth syndrome, Friedreich ataxia

## Abstract

Mitochondria are found in all nucleated human cells and perform various essential functions, including the generation of cellular energy. Mitochondria are under dual genome control. Only a small fraction of their proteins are encoded by mitochondrial DNA (mtDNA), whereas more than 99% of them are encoded by nuclear DNA (nDNA). Mutations in mtDNA or mitochondria-related nDNA genes result in mitochondrial dysfunction leading to insufficient energy production required to meet the needs for various organs, particularly those with high energy requirements, including the central nervous system, skeletal and cardiac muscles, kidneys, liver, and endocrine system. Because cardiac muscles are one of the high energy demanding tissues, cardiac involvement occurs in mitochondrial diseases with cardiomyopathies being one of the most frequent cardiac manifestations found in these disorders. Cardiomyopathy is estimated to occur in 20–40% of children with mitochondrial diseases. Mitochondrial cardiomyopathies can vary in severity from asymptomatic status to severe manifestations including heart failure, arrhythmias, and sudden cardiac death. Hypertrophic cardiomyopathy is the most common type; however, mitochondrial cardiomyopathies might also present as dilated, restrictive, left ventricular non-compaction, and histiocytoid cardiomyopathies. Cardiomyopathies are frequent manifestations of mitochondrial diseases associated with defects in electron transport chain complexes subunits and their assembly factors, mitochondrial transfer RNAs, ribosomal RNAs, ribosomal proteins, translation factors, mtDNA maintenance, and coenzyme Q_10_ synthesis. Other mitochondrial diseases with cardiomyopathies include Barth syndrome, Sengers syndrome, *TMEM70*-related mitochondrial complex V deficiency, and Friedreich ataxia.

## Introduction

Metabolic disorders account for a minority of causes of cardiomyopathies. However, diagnosing a metabolic disease as a cause for cardiomyopathy can have prognostic and therapeutic implications. Major groups of metabolic disorders associated with cardiomyopathy include organic acidemias (e.g., propionic acidemia), fatty acid oxidation defects (e.g., very long chain acyl CoA dehydrogenases deficiency), lysosomal storage diseases (e.g., Fabry disease), glycogen storage diseases (e.g., Pompe disease), congenital disorders of glycosylation, and mitochondrial disorders (1).

Mitochondrial diseases are a clinically and genetically heterogeneous group of disorders that result from dysfunction of the mitochondrial respiratory chain, which is responsible for the generation of most cellular energy (2, 3). Because cardiac muscles are one of the high energy demanding tissues, cardiac involvement occurs in large number of mitochondrial diseases. The most frequent cardiac manifestations of mitochondrial diseases are cardiomyopathies. Arrhythmias and conduction defects, pulmonary hypertension, pericardial effusion, dilated aortic root, and coronary heart disease can also be seen in mitochondrial diseases (4, 5).

In this article, we review normal mitochondrial structure and function, pathogenesis of mitochondrial diseases, clinical aspects of mitochondrial cardiomyopathies, mitochondrial diseases frequently associated with cardiomyopathies, and diagnosis and management of mitochondrial cardiomyopathies.

## Normal Mitochondrial Structure and Function

Mitochondria are found in all nucleated human cells each of which typically contains in its cytoplasm several hundred mitochondria depending on the energy needs for the tissue. Mitochondria are composed of two bilayer membranes that create two distinct compartments: an intermembrane space and a matrix space within the inner membrane. The mitochondrial outer membrane is smooth, whereas the inner mitochondrial membrane is highly folded, forming structures called cristae. The large surface area of the inner mitochondrial membrane accommodates energy-generating multipolypeptide enzyme complexes called respiratory chain or electron transport chain (ETC) complexes (2).

Approximately 1,500 proteins are involved in maintaining mitochondrial structure and function; however, <1% are encoded by mitochondrial DNA (mtDNA), while more than 99% of mitochondrial proteins are encoded by nuclear DNA (nDNA). Therefore, mitochondria are under dual genome control. Each mitochondrion contains mtDNA in the form of a multicopy, 16.6 kb circular double-stranded DNA. The mtDNA encodes 13 essential polypeptides for the ETC complexes and 24 different RNAs, including 2 ribosomal RNAs (rRNAs) and 22 transfer RNAs (tRNAs) (3, 6). The remaining ETC complexes subunits, as well as proteins needed to assemble the ETC complexes (assembly factors), maintain mtDNA, and transport molecules across the mitochondrial membranes, are encoded by nDNA, synthesized on cytoplasmic ribosomes, and imported into mitochondria (7). Unlike nDNA, which replicates with each cell division, mtDNA replicates continuously and independently of cell division. Two nDNA-encoded enzymes play major roles in mtDNA replication: DNA polymerase gamma that functions in replication and repair of mtDNA, and the twinkle protein that serves the function of a DNA helicase that is required for mtDNA replication (8). Transcription of mtDNA produces a polycistronic precursor RNA that is then processed to produce individual mRNA, tRNA, and rRNA molecules. The nDNA-encoded mitochondrial RNA polymerase and mitochondrial transcriptions factors are needed for the mitochondrial transcription process (9). The mRNAs for the 13 mtDNA-encoded proteins are translated on mitochondrial ribosomes. Mitochondrial tRNAs and rRNAs are required for this process in addition to several nDNA-encoded proteins, including mitochondrial ribosomal proteins and mitochondrial translation factors (9). The nDNA-encoded mitochondrial polypeptides are synthesized on cytosolic ribosomes and transported into the mitochondria *via* mitochondrial protein import systems, including the translocase of the outer membrane (TOM) and translocase of the inner membrane (TIM) complexes (7, 10).

**Figure 1:**
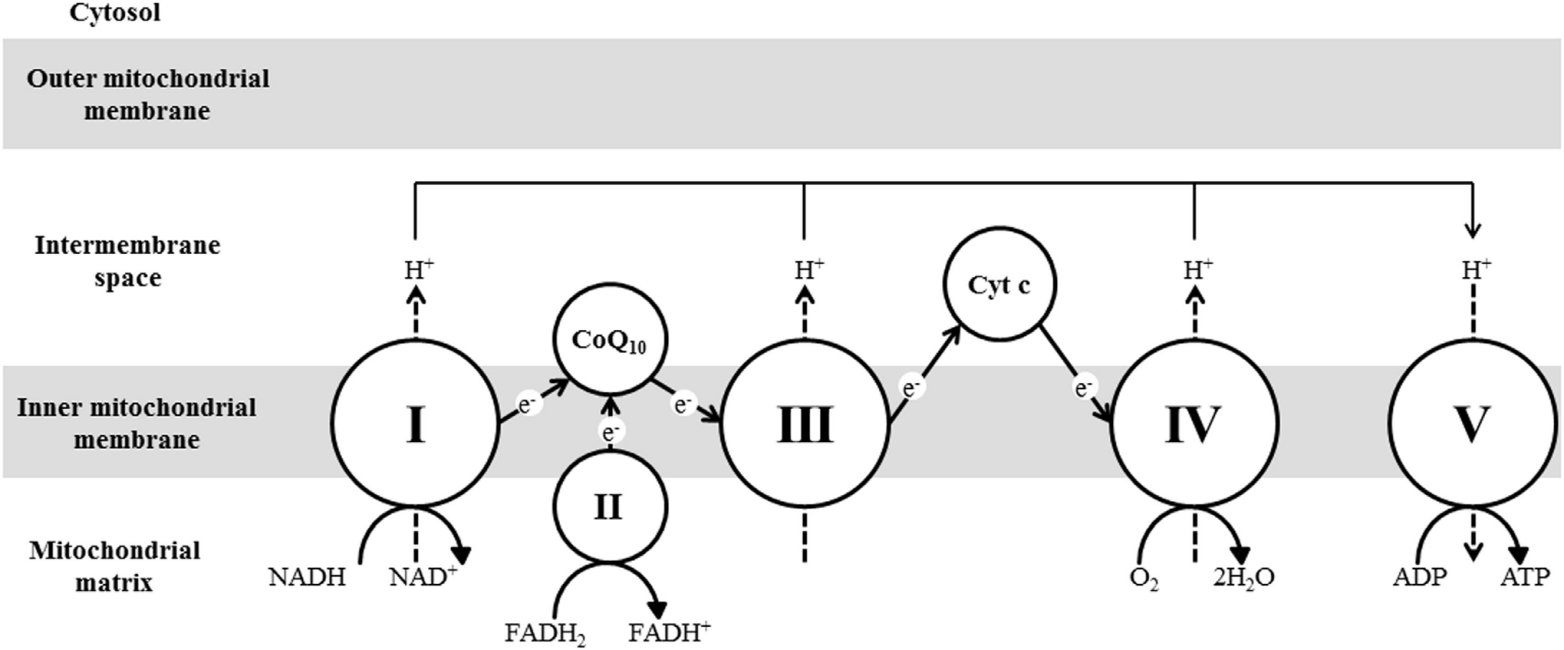
**A diagram showing electron transfer along the mitochondrial ETC complexes, hydrogen pumping across the inner mitochondrial membrane, and ATP synthesis**.

Mitochondria perform various essential functions, including the generation of most of the energy needed by cells in the form of ATP in a process called oxidative phosphorylation (OXPHOS) carried out by the ETC complexes in the inner mitochondrial membrane. Complexes I, II, III, and IV make up the ETC, whereas complex V is the ATP synthase. Hydrogen atoms generated from different catabolic pathways bind to nicotinamide adenine dinucleotide (NAD^+^) and flavin adenine dinucleotide (FAD) to yield NADH and FADH_2_, respectively. NADH is oxidized by complex I (NADH dehydrogenase), and the electrons are transported through flavin mononucleotide (FMN) and multiple iron–sulfur (Fe–S) centers in complex I until they are transferred to coenzyme Q_10_ (CoQ_10_). CoQ_10_ also accepts hydrogen atoms from FADH_2_ generated by β-oxidation and the TCA enzyme succinate dehydrogenase (complex II). Electrons are subsequently transferred from CoQ_10_ to complex III (*bc*1 complex) within which the electrons move through cytochrome *b*, cytochrome *c*1, and the Fe–S components. The electrons are then transferred from complex III to cytochrome *c*, which transfers the electrons to complex IV (cytochrome *c* oxidase). Within this complex, the electrons are transferred through copper centers and cytochromes *a* and *a*3 and ultimately combine with O_2_ to generate H_2_O. The energy that is released during electron transfer is used to pump protons from inside the mitochondrial matrix across the inner mitochondrial membrane into the intermembrane space through complexes I, III, and IV. The resulting electrochemical gradient forces protons to move back through a proton channel in complex V (ATP synthase), which utilizes this energy in synthesizing ATP. The ETC complexes are multipolypeptides encoded by both mtDNA and nDNA except for complex II, which is encoded entirely by nDNA (11) (Figure 1).

## Mitochondrial Dysfunction and Diseases

Mutations in mtDNA or mitochondria-related nDNA genes result in mitochondrial dysfunction leading to mitochondrial diseases (12). Defects in mtDNA can be either point mutations or rearrangements. Point mutations in mtDNA can affect protein-encoding genes or genes encoding tRNA or rRNA. These mutations are maternally inherited and typically associated with very variable phenotypes. Rearrangements of mtDNA include deletions and duplications that differ in size and position but typically encompass several genes. These rearrangements are usually sporadic arising *de novo* but can be maternally inherited (13). Mutations in nDNA genes are inherited in an autosomal recessive, autosomal dominant, or X-linked manner. Mitochondrial dysfunction can result from mutations in nDNA genes encoding ETC complexes subunits or their assembly factors (11), mitochondrial import complexes (10), mitochondrial ribosomal proteins and translational factors (14), and CoQ_10_ biosynthesis enzymes (15). The mtDNA is maintained by a group of nDNA-encoded proteins that function either in mitochondrial deoxyribonucleoside triphosphate (dNTP) synthesis or mtDNA replication. Mutations in any of these genes result in depletion of the mitochondrial dNTP pool or impaired mtDNA replication, leading to severe reduction in mtDNA content (mtDNA depletion). Inadequate amount of mtDNA results in impaired synthesis of key subunits of ETC complexes (16). Finally, Fe–S clusters are ubiquitous cofactors that are composed of iron and inorganic sulfur. These clusters are important prosthetic groups that are required for the function of proteins involved in various activities, including electron transport in ETC complexes. Defects in the process of Fe–S clusters can result in impaired ETC activity and mitochondrial dysfunction (17).

Defects in mtDNA- or nDNA-encoded mitochondrial proteins result in mitochondrial respiratory chain dysfunction leading to impaired OXPHOS and inability to generate sufficient energy to meet the needs for various organs, particularly those with high energy demand, including the central nervous system, skeletal and cardiac muscles, kidneys, liver, and endocrine system (2, 3). Additionally, due to the impaired OXPHOS, NADH cannot be utilized and the NADH:NAD ratio increases, which results in the inhibition of the TCA cycle. Pyruvate, produced through glycolysis, is increased due to the TCA cycle inhibition. Both elevated pyruvate and NADH:NAD ratio result in shifting the equilibrium of lactate dehydrogenase toward the production of lactate from pyruvate. Lactate can accumulate, causing systemic acidosis. Lactic acidosis is among one of the common features of mitochondrial disorders (6).

In addition to ATP deficiency, consequences of mitochondrial dysfunction include aberrant calcium handling, excessive reactive oxygen species (ROS) production, apoptosis dysregulation, and nitric oxide (NO) deficiency all of which contribute to the pathogenesis of mitochondrial diseases (18). During OXPHOS, a small part of oxygen is partially reduced and converted to ROS (superoxide and hydrogen peroxide). Under normal conditions, ROS can be scavenged by various enzymes, including the mitochondrial superoxide dismutase and glutathione peroxidase (19, 20). ROS, whose generation is enhanced as a result of OXPHOS blockade, can irreversibly modify many cellular macromolecules leading to cellular toxicity. Increased ROS production in mitochondrial diseases can result in protein, lipid, and DNA damage, which can potentially lead to further cellular damage and dysfunction (19, 20). One of the mitochondrial functions is calcium buffering. In addition, mitochondrial ATP production is needed to fuel calcium pumps in the plasma membrane and endoplasmic reticulum. Therefore, mitochondrial dysfunction can result in aberrant calcium handling. This model could contribute to the frequent involvement of muscle and nerve tissues in mitochondrial diseases, since these cells rely heavily on ATP and on fluctuating levels of intracellular calcium (21, 22). Mitochondria are also major regulators of apoptosis. In response to several intracellular stress conditions, supermolecular channels called mitochondrial permeability transition pores open resulting in increased mitochondrial inner membrane permeability. Apoptosis is initiated when the inner mitochondrial membrane becomes permeable leading to the release of several toxic mitochondrial proteins into the cytosol, including cytochrome *c*. These proteins activate latent forms of caspases, resulting in the execution of apoptosis. Therefore, excessive cell loss can contribute to the pathology in mitochondrial diseases (23). Finally, there is growing evidence that NO deficiency occurs in mitochondrial diseases and can play a major role in the pathogenesis of several complications observed in these diseases, including stroke-like episodes, myopathy, diabetes, and lactic acidosis. NO deficiency in mitochondrial disorders is multifactorial in origin, including impaired NO production and postproduction sequestration (24).

The mtDNA in cells can be identical (homoplasmy) or a mixture of two or more types (heteroplasmy). Some mtDNA mutations affect all copies of the mtDNA (homoplasmic mutations), while most of the mutations are present in only some copies of mtDNA and cells harbor a mixture of mutant and normal mtDNA (heteroplasmic mutations) (25). When cell divides, the mitochondria are distributed in a stochastic process in daughter cells. Therefore, when a cell harboring a heteroplasmic mtDNA mutation divides, it is a matter of chance whether the mutant mtDNAs will be partitioned into one daughter cell or another. Therefore, over time, the percentage of mutant mtDNAs can differ in different tissues and organs. This process, which is called replicative segregation, explains why the heteroplasmy percentage of mutant mtDNA may vary among organs and tissues within the same individual. The different tissues and organs rely on mitochondrial energy to different extents. As the percentage of mutated mtDNA increases, energy production declines. When the proportion of mutant mtDNA crosses a critical threshold level, the impaired energy production will result in organ dysfunction and clinical manifestations. The threshold level varies among different organs and tissues depending on their energy requirement (26). The replicative segregation and different organ threshold levels can explain in part the varied clinical phenotypes observed in individuals with mtDNA mutations. On the other hand, the clinical phenotypes of nDNA-related mitochondrial diseases are typically more homogenous than the mtDNA-related disease, as all the mitochondria are similarly affected (3).

Mitochondrial disorders are not uncommon with a minimum prevalence of 1 in 5,000 (12). Mitochondria are essential components of all nucleated cells. Therefore, mitochondrial dysfunction affects many organs, particularly those with high energy requirements. Insufficient energy for various organs results in multi-organ dysfunction and the variable manifestations observed in mitochondrial diseases, including epilepsy, intellectual disability, skeletal and cardiac myopathies, hepatopathies, endocrinopathies, and nephropathies (2, 3, 6). Although the vast majority of mitochondrial diseases involve multiple organ systems, some mitochondrial diseases may affect a single organ (e.g., Leber hereditary optic neuropathy, and non-syndromic sensorineural hearing loss) (2). Mitochondrial diseases can begin at any age. Many patients with mitochondrial diseases display a cluster of clinical features that fall into a discrete clinical syndrome such as Kearns–Sayre syndrome, mitochondrial encephalomyopathy with lactic acidosis and stroke-like episodes (MELAS), myoclonic epilepsy with ragged-red fibers (MERRF), neurogenic weakness with ataxia and retinitis pigmentosa (NARP), mitochondrial neurogastrointestinal encephalopathy (MNGIE), and Alpers syndrome. However, there is often considerable clinical variability, and many affected individuals do not fit into one particular syndrome (2, 3, 6).

## Clinical Aspects of Mitochondrial Cardiomyopathies

Mitochondrial cardiomyopathy can be described as a myocardial disorder characterized by abnormal myocardial structure and/or function secondary to genetic defects resulting in the impairment of the mitochondrial respiratory chain, in the absence of concomitant coronary artery disease, hypertension, valvular disease, and congenital heart disease (27). Cardiomyopathy is estimated to occur in 20–40% of children with mitochondrial diseases (5, 28). Therefore, screening for cardiomyopathy is a standard part of the management of children and adults with known or suspected mitochondrial disease (29).

Mitochondrial cardiomyopathies can vary in severity from asymptomatic status to severe manifestations, including heart failure, arrhythmias, and sudden cardiac death. Cardiac manifestations can be precipitated or worsen during metabolic decompensation episodes that are often caused by stressors, such as febrile illnesses or surgery, and can be accompanied by acute heart failure (27). It has been reported that mortality in children with mitochondrial diseases is significantly higher in those with cardiomyopathy than in those without (28). The clinical manifestations of mitochondrial cardiomyopathies are often accompanied by other manifestations of the multi-organ involvement of mitochondrial diseases. On the other hand, mitochondrial cardiomyopathy can occur in the absence of known mitochondrial disease, of which it may be the first or the sole clinical manifestation (29).

Hypertrophic cardiomyopathy is the most common form; however, mitochondrial cardiomyopathies might also present as dilated, restrictive, left ventricular non-compaction, and histiocytoid cardiomyopathies (4). Hypertrophic cardiomyopathy is the most frequent cardiac manifestation in mitochondrial diseases and can occur in more than 50% of individuals with mitochondrial cardiomyopathies (5). It can be detected as early as the antenatal period and may be the only manifestation of a mitochondrial disease or a part of a multi-organ disease. Obstructive hypertrophic cardiomyopathy rarely occurs, but hypertrophic cardiomyopathy frequently develops into systolic dysfunction followed by decompensation and dilatation of the left ventricle (30). Dilated cardiomyopathy, which can be primary or secondary following hypertrophic cardiomyopathy, occurs less frequently than hypertrophic cardiomyopathy, whereas restrictive cardiomyopathy is a rare manifestation of mitochondrial diseases (31). Although left ventricular non-compaction cardiomyopathy is also a rare finding in mitochondrial diseases, among individuals with non-compaction, mitochondrial diseases are highly prevalent. Left ventricular non-compaction cardiomyopathy is generally more frequent in males and tends to develop during pregnancy in females. Occasionally, it may disappear during the disease course in some individuals with mitochondrial diseases (32). Histiocytoid cardiomyopathy (Purkinje fiber dysplasia) is histologically characterized by morphological and functional abnormalities of cardiomyocytes and Purkinje cells with a cytoplasm like in histiocyte foam cells, which contain glycogen and lipids. It has been reported exclusively in individuals with mitochondrial diseases (33).

## Mitochondrial Diseases Frequently Associated with Cardiomyopathies

Cardiomyopathies are frequent manifestations of mitochondrial diseases associated with defects in ETC complexes subunits and their assembly factors, mitochondrial tRNAs, rRNAs, ribosomal proteins, translation factors, mtDNA maintenance, and CoQ_10_ synthesis. Other mitochondrial diseases with cardiomyopathies include Barth syndrome and other 3-methylglutaconic aciduria disorders, and Friedreich ataxia (Table 1).

**Table 1:** **Mitochondrial diseases frequently associated with cardiomyopathies**.

Mitochondrial diseases	Genes	Clinical manifestations
**ETC complexes deficiencies**		
Complex I deficiency	Subunit mtDNA genes: *MTND1, MTND2, MTND4, MTND5*, and *MTND6* Subunit nDNA genes: *NDUFV1, NDUFV2, NDUFS1, NDUFS2, NDUFS3, NDUFS4, NDUFS6, NDUFS7, NDUFS8, NDUFA2, NDUFA11, NDUFAF3, NDUFA10, NDUFB3, NDUFB9*, and *NDUFA1* Assembly genes: *NDUFAF2, NDUFAF4, NDUFAF5, NUBPL, NDUFAF1, FOXRED1*, and *ACAD9*	Hypertrophic cardiomyopathy Growth failure Developmental delay Epilepsy Ataxia Weakness Spasticity Leukoencephalopathy Macrocephaly Sensorineural deafness Hepatic dysfunction Lactic acidosis Hypoglycemia
Complex II deficiency	Subunit genes: *SDHA* and *SDHD*	Hypertrophic, dilated, and non-compaction cardiomyopathies Growth failure Developmental delay Weakness Spasticity Ataxia Epilepsy Leukodystrophy Contractures Ophthalmoplegia Pigmentary retinopathy Optic atrophy Lactic acidosis
Complex III deficiency	*MTCYB*	Hypertrophic, dilated, and histiocytoid cardiomyopathies Growth failure Exercise intolerance Optic atrophy Stroke-like episodes Epilepsy Lactic acidosis Hypoglycemia
Complex IV deficiency	Subunit mtDNA genes: *MTCO1, MTCO2*, and *MTCO3* Subunit nDNA genes: *COX6B1* Assembly factors: *COX10, COX14, COX15, COX20, SCO1, SCO2, COA3*, and *COA5*	Dilated, hypertrophic, and histocytoid cardiomyopathies Growth failure Developmental delay Ataxia Epilepsy Hypotonia Sensorineural hearing loss Optic atrophy Pigmentary retinopathy Liver dysfunction Renal tubulopathy Lactic acidosis

**Mitochondrial tRNA genes**		
MERRF (myoclonic epilepsy with ragged-red fibers) syndrome	*MTTK*	Dilated and histiocytoid cardiomyopathy Epilepsy Ataxia Weakness Sensorineural hearing loss Short stature Lactic acidosis
MELAS (mitochondrial encephalomyopathy, lactic acidosis, and stroke-like episodes) syndrome	*MTTL1*	Hypertrophic cardiomyopathy Muscle weakness Stroke-like episodes Dementia Epielpsy Sensorineural hearing loss Lactic acidosis Diabetes Short stature

**Mitochondrial DNA depletion**		
Mitochondrial neurogastrointestinal encephalopathy syndrome (MNGIE)	*TYMP*	Hypertrophic cardiomyopathy Gastrointestinal dysmotility Cachexia Ptosis Ophthalmoplegia Hearing loss Peripheral neuropathy Leukoencephalopathy

**CoQ10 deficiency**		
Coenzyme Q10 deficiency	*COQ2, COQ4, COQ6, COQ7, COQ9, ADCK3, PDSS1*, and *PDSS2*	Hypertrophic cardiomyopathy Growth failure Developmental delay Weakness Epilepsy Ataxia Pigmentary retinopathy Sensorineural hearing loss Liver dysfunction Renal impairment Pancytopenia Lactic acidosis

**3-Methylglutaconic acidurias**		
3-Methylglutaconic aciduria type II (Barth syndrome)	*TAZ*	Non-compaction, dilated, and hypertrophic cardiomyopathies Growth failure Weakness Arrhythmias Neutropenia
3-Methylglutaconic aciduria, type V (dilated cardiomyopathy and ataxia syndrome)	*DNAJC19*	Dilated and non-compaction cardiomyopathies Growth failure Ataxia Testicular dysgenesis Anemia
Mitochondrial complex V deficiency	*TMEM70*	Hypertrophic cardiomyopathy Growth failure Developmental delay Hypotonia Ataxia Epilepsy Leukodystrophy Distinctive facial features Lactic acidosis Hyperammonemia
Sengers syndrome	*AGK*	Hypertrophic cardiomyopathy Growth failure Cataracts Hypotonia Weakness Lactic acidosis

**Defects in iron–sulfur cluster**		
Friedreich ataxia	*FXN*	Hypertrophic cardiomyopathy Ataxia Dysarthria Peripheral sensory neuropathy Diabetes mellitus

Complex I deficiency, which is clinically and genetically heterogeneous, can present with hypertrophic cardiomyopathy that might be isolated or associated with multi-organ disease. Cardiomyopathy has been reported with mutations in mitochondrial (e.g., *MTND1* and *MTND5*) and nuclear (e.g., *NDUFS2, NDUFV2*, and *NDUFA2*) genes encoding complex I subunits, and nuclear genes that encode complex I assembly factors (e.g., *ACAD9* and *NDUFAF1*) (34, 35). Complex II is entirely encoded by nDNA, and its deficiency has been reported in individuals with hypertrophic, dilated, and non-compaction cardiomyopathies who carried mutations in complex II subunits genes (*SDHA* and *SDHD*) (36, 37). Complex III deficiency can also cause cardiomyopathy that can either be isolated or accompanied with multi-organ involvement. Hypertrophic, dilated, and histiocytoid cardiomyopathies were reported in individuals with complex III deficiency and mutations in the *MTCYB* gene encoding cytochrome *b* (38–40). Dilated, hypertrophic, and histocytoid cardiomyopathies have been reported in complex IV deficiencies associated with mutations in complex IV subunit genes (*COX6B1, MTCO2*, and *MTCO3*) and complex IV assembly factors genes (*SURF1* and *SCO2*) (41, 42).

Mutations in several mitochondrial tRNA genes (e.g., *MTTK* causing MERRF syndrome and *MTTL1* causing MELAS syndrome) have been reported with multi-organ mitochondrial diseases or isolated cardiomyopathies. Cardiomyopathies associated with pathogenic variants in genes encoding mitochondrial tRNAs are usually hypertrophic, but can also be dilated or histiocytoid cardiomyopathy (29, 43). Hypertrophic cardiomyopathy has been reported with mutations in the mitochondrial 16S rRNA gene (*MTRNR2*) and restrictive cardiomyopathy with the m.1555A>G mutation in the mitochondrial 12S rRNA gene (*MTRNR1*) that is typically associated with aminoglycoside-induced hearing loss (44, 45). Mutations in genes coding mitochondrial ribosomal proteins (e.g., *MRPL3* and *MRPL44*) can cause hypertrophic cardiomyopathy accompanied by multi-organ disease (46, 47). Mutations in *TSFM*, encoding a mitochondrial translation elongation factor, can be associated with hypertrophic or dilated cardiomyopathy associated with multi-organ disease (48).

Mitochondrial neurogastrointestinal encephalopathy (MNGIE) syndrome is an mtDNA depletion syndrome caused by deficiency of thymidine phosphorylase, resulting in imbalances in mitochondrial nucleotide pools. Clinical features of MNGIE include progressive gastrointestinal dysmotility and cachexia, ptosis, ophthalmoplegia, hearing loss, demyelinating peripheral neuropathy, and leukoencephalopathy. Cardiac manifestations are usually asymptomatic ventricular hypertrophy and bundle branch block (49, 50).

Defects in CoQ_10_ biosynthesis result in primary CoQ_10_ deficiency which is a phenotypically and genetically heterogeneous condition with various clinical presentations, including encephalomyopathy, isolated myopathy, cerebellar ataxia, and nephrotic syndrome. Hypertrophic cardiomyopathy has been reported with mutations in genes involved in CoQ_10_ biosynthesis (*COQ2, COQ4*, and *COQ9*) (51, 52).

Barth syndrome is an X-linked disorder characterized by cardiomyopathy, skeletal myopathy, growth retardation, neutropenia, and increased urinary levels of 3-methylglutaconic acid. It is caused by mutations in the *TAZ* gene that codes for tafazzin, a phospholipid transacylase located in the inner mitochondrial membrane and plays an important role in the remodeling of cardiolipin. Cardiomyopathies are commonly left ventricular non-compaction and dilated cardiomyopathies, whereas hypertrophic cardiomyopathy appears to be less common. Other cardiac manifestations of Barth syndrome are arrhythmia (including supraventricular and ventricular tachycardia) and sudden death (53, 54).

Barth syndrome is one of a small group of disorders characterized by 3-methylglutaconic aciduria as a discriminative feature, where excretion of 3-methylglutaconic acid is significant and consistent. Other disorders in this group that might be associated with cardiomyopathy are caused by mutations in *DNAJC19, TMEM70*, and *AGK* (55). 3-Methylglutaconic aciduria associated with *DNAJC19* mutations (dilated cardiomyopathy and ataxia syndrome), results from deficient mitochondrial protein import and is characterized by dilated cardiomyopathy or left ventricular non-compaction, non-progressive cerebellar ataxia, testicular dysgenesis, and growth failure (56). Mutations in *TMEM70* (mitochondrial complex V deficiency), encoding a protein involved in the insertion of ATP synthase (complex V) into the mitochondrial membrane, result in multi-organ mitochondrial disease with hypertrophic cardiomyopathy (57). Sengers syndrome, caused by mutations in *AGK*, might also be accompanied by 3-methylglutaconic aciduria and is characterized by hypertrophic cardiomyopathy, cataracts, myopathy, exercise intolerance, and lactic acidosis. The *AGK* gene product is an acylglycerol kinase and is involved in the assembly of ANT1, a mitochondrial adenine nucleotide transporter (58).

Friedreich ataxia is an autosomal recessive neurodegenerative disorder caused by mutations of *FXN*, which encodes frataxin, a mitochondrial iron-binding protein involved in the synthesis of the Fe–S clusters required by the ETC complexes. The clinical presentation includes progressive ataxia after the teenage years, dysarthria, loss of lower limb reflexes, peripheral sensory neuropathy, and diabetes mellitus. The cardiac manifestations include hypertrophic cardiomyopathy (59, 60).

## Diagnosis and Management of Mitochondrial Cardiomyopathies

The diagnosis of mitochondrial diseases is based on clinical recognition, biochemical screening, histopathological studies, functional assays, and molecular genetic testing. Due to the multi-organ involvement in the majority of mitochondrial diseases, evaluation of these diseases should include a systematic screening for all the targeted organs, e.g., neuroimaging, hearing assessment, ophthalmologic examination, liver function test, and serum creatinine phosphokinase (2). Biochemical screening tests for mitochondrial disorders include the determination of plasma lactate, blood glucose, urine organic acids, and plasma amino acids. Although lactic acidemia is a common biochemical feature of many mitochondrial disorders, it is neither specific nor sensitive (61). Hypoglycemia can be seen in children with mitochondrial diseases and urine organic acid analysis can show non-specific findings, including elevated lactate, ketone bodies, and TCA intermediates. A plasma amino acid profile may show elevation in plasma alanine level which reflects lactic acidemia and branched-chain amino acids which are catabolized in mitochondria (18).

Analysis of a fresh skeletal muscle biopsy is considered the gold standard in the diagnosis of mitochondrial disorders. The histology of affected muscles typically shows ragged-red fibers, which can be demonstrated using the modified Gomori trichrome stains, and contains peripheral and intermyofibrillar accumulation of abnormal mitochondria. Examining the muscle under an electron microscopy can demonstrate mitochondrial proliferation and abnormal mitochondrial morphology in mitochondrial myopathies. Histochemical staining for different ETC complexes can be used to estimate the severity and heterogeneity of ETC complexes deficiencies in the muscle tissue (3). Mitochondrial function can be assessed by measuring the enzymatic activity of different ETC complexes using a spectrophotometric methodology that utilizes specific electron acceptors and donors. This assessment is usually carried out on skeletal muscle, skin fibroblast, or liver tissue (11). Mitochondrial function can also be assessed using the extracellular flux analyzer, Seahorse instrument, which can simultaneously measure mitochondrial respiration and glycolysis (62). Cardiac muscle biopsy is more invasive and can be performed in a patient with rapid disease progression or when biochemical testing in fibroblasts and skeletal muscle and molecular testing have not led to a conclusive diagnosis (29).

Molecular testing includes assessment of mtDNA content and DNA sequencing. Increased mtDNA content suggests a compensatory mechanism due to deficient mitochondrial function, whereas reduced mtDNA content implies defects in mtDNA biosynthesis, leading to mtDNA depletion. Measurement of mtDNA copy number is performed by real-time quantitative polymerase chain reaction using a mtDNA probe and a unique nuclear gene reference (63). Variable DNA sequencing options are available. If the clinical features of a mitochondrial disease are consistent with a recognizable syndrome, the mtDNA or nDNA gene known to be responsible for that syndrome can be tested to confirm the diagnosis. If a maternally inherited mitochondrial disease is suspected, the whole mtDNA can be sequenced. When genetically heterogeneous nDNA gene-related mitochondrial disease (e.g., mtDNA depletion syndromes) panel tests that include the known genes associated with such disease can be helpful. Next-generation massively parallel sequencing, which allows simultaneous sequencing of multiple genes at high coverage and low cost, has been widely used method for these gene panels. When the clinical picture is not consistent with a disease related to a specific gene or group of genes, a more extensive panel that includes all the known nDNA-related mitochondrial genes or whole exome or genome sequencing methodology can be considered (64, 65).

Currently, there are no satisfactory therapies available for mitochondrial disorders. Treatment remains largely symptomatic and does not significantly alter the course of the disease. Several cofactor supplementations have been tried with limited data supporting their benefits for most of them (6). So far, the only mitochondrial cardiomyopathies with an effective and specific metabolic treatment are those caused by CoQ_10_ deficiency. CoQ_10_ (ubiquinone) supplementation for patients with CoQ_10_ deficiency results in restoring the electron flow and a dramatic improvement in clinical manifestations associated with CoQ_10_ deficiency (66).

Heart transplantation was reported to be performed in 14% of patients with Barth syndrome (53). With respect to other mitochondrial diseases, although multi-organ diseases are considered a relative contraindication for solid organ transplantation, heart transplantation might be successful when clinical expression is limited to the myocardium or manifestations outside the heart are mild and appear non-progressive (29).

Ongoing clinical trials for potential treatment of mitochondrial diseases include the use of Bendavia, a mitochondrial permeability transition pore inhibitor, RTA 408, a potent activator of Nrf2 which is a regulator of cellular resistance to oxidants, and cysteamine bitartrate, an antioxidant (67) (http://Clinicaltrials.gov).

## Conclusion

Hypertrophic, dilated, non-compaction, and histiocytoid cardiomyopathies can be the only feature or part of multi-organ mitochondrial diseases. Cardiomyopathies occur in approximately one-third of children with mitochondrial diseases and increase the mortality in these children. Therefore, screening for cardiomyopathy is a standard part of the management of individuals with known or suspected mitochondrial disease. Diagnosing mitochondrial diseases remains challenging in many cases and treatment remains largely symptomatic, as there are no satisfactory therapies available that significantly alter the course of the disease. Therefore, a lot of work is still need to be done to facilitate early diagnosis through discovering new disease biomarkers and novels genes involved in mitochondrial function and to find new treatment strategies that can restore the mitochondrial function.

## Author Contributions

Dr. AE-H has written the initial draft. Dr. FS has reviewed and modified the draft.

## Conflict of Interest Statement

The authors declare that the research was conducted in the absence of any commercial or financial relationships that could be construed as a potential conflict of interest.
